# Antimicrobial Active Clothes Display No Adverse Effects on the Ecological Balance of the Healthy Human Skin Microflora

**DOI:** 10.5402/2011/369603

**Published:** 2011-04-04

**Authors:** Dirk Hoefer, Timo R. Hammer

**Affiliations:** Institute for Hygiene and Biotechnology, Hohenstein Institutes, Schloss Hohenstein, 74357 Boennigheim, Germany

## Abstract

The progressive public use of antimicrobial clothes has raised issues concerning skin health. A placebo-controlled side-to-side study was run with antimicrobial clothes versus fabrics of similar structure but minus the antimicrobial activity, to evaluate possible adverse effects on the healthy skin microflora. Sixty volunteers were enrolled. Each participant received a set of form-fitting T-shirts constructed in 2 halves: an antibacterial half, displaying activities of 3–5 log-step reductions due to silver-finishes or silver-loaded fibres and a nonantibacterial control side. The microflora of the scapular skin was analyzed weekly for opportunistic and pathogenic microorganisms over six weeks. The antibacterial halves did not disturb the microflora in number or composition, whereas a silver-containing deodorant displayed a short-term disturbance. Furthermore, parameters of skin morphology and function (TEWL, pH, moisture) did not show any significant shifts. In summary, antimicrobial clothes did not show adverse effects on the ecological balance of the healthy skin microflora.

## 1. Introduction

Originally, antimicrobial substances have been used in textiles to prevent rotting, especially under tropical climate conditions. Nowadays, consumers' attitude towards hygiene and active lifestyle has created a rapidly increasing market for antimicrobial consumer goods; hence, the application of antimicrobial agents is extended to clothes used in outdoor, health care sector, sport, and leisure. 

The majority of fabrics use silver ions as the active antimicrobial agent [1]. Beside silver, quaternary ammonium compounds, polyhexamethylene biguanides, triclosan, or chitosan are also used. Antimicrobial agents can be applied to the textile substrates as a finish by exhaust, pad-dry-cure, coating, spray, and foam techniques, or the substances can be applied by directly adding into the fibre spinning dope [2]. Manufacturers claim that the antimicrobial effect is restricted more or less to the fibre surface, but mostly the amount of biocide released onto the skin from each product is unknown. 

In dermatology, antimicrobials are mainly used as liquids to eliminate pathogens in skin antisepsis and disinfection. The application of therapy-enhancing antimicrobial fabrics in dermatology came up in 2006, when Gauger et al. used form-fitting antimicrobial textiles, based on silver-coated yarns in the treatment of atopic dermatitis [3]. In this double-blind, placebo-controlled trial with 68 atopic dermatitis patients, they were able to show that antimicrobial fabrics, worn for 2 weeks tightly on the skin, may reduce the nonphysiological colonization of the patients skin with the microorganism *Staphylococcus aureus* [4, 5]. Subsequently similar studies confirmed that antimicrobial cloth at least have influence on the pathological skin flora of atopic dermatitis skin and thus may support or reconstitute physiological functions [6–8]. Whether an influence on the physiological skin flora on skin of healthy subjects occurs has not been addressed so far. 

In contrast to therapy-enhancing textiles, which support physiological or healing functions, the public use of antimicrobial cloth as a consumer good should not pose any risk to the human health under normal or foreseeable use [8–13]. The question of such health risks is important for the increasing number of people using antimicrobial cloth especially in sport and leisure activities, who wish to feel clean and safe or to control malodour. The main concerns with the regular use of topical antimicrobial substances on skin comprise the development of irritant and allergic dermatitis [14] as well as disturbances in the ecological balance between the host (transient) and the normal (resident) microflora. Since most studies on the impact of antimicrobial agents on normal microflora have been carried out on the intestinal flora [15], less is known on the effects on the human skin microflora [16], although the skin microbiota provides an important barrier against the colonization of potentially pathogenic microorganisms and against overgrowth of already present opportunistic microorganisms [15]. Proposed beneficial roles also include further processing of skin proteins, free fatty acids, and sebum [17].

Adverse effects of antimicrobial clothes, especially form-fitting sport and leisure underwear, on the ecological balance of the human skin microflora, are poorly studied. We therefore investigated in this study, whether silver-finished and silver-loaded antimicrobial fabrics lead to changes in the physiological human skin microflora of healthy subjects under usual use. To address this question, a placebo-controlled right/left-intraindividual pre-/post-comparison trial with 60 volunteers was performed over a period of 6 weeks. Antimicrobial fabrics, provided with a strong antimicrobial activity according to ISO 20743, were used in this long-term wear trial and compared with the short-term application of an antibacterial silver-containing deodorant. Furthermore, we evaluated the effect of the antimicrobial fabrics on skin physiological parameters. In particular, transepidermal water loss (TEWL), stratum corneum hydration (corneometry) and skin surface pH (pHmetry) were objectively used to monitor the skin barrier functions, in order to look for the advent of irritations or secondary effects of a changing microbial composition of the skin microflora.

## 2. Materials and Methods

### 2.1. Subjects

In all, 60 healthy, Caucasian volunteers, 30 female and 30 male (mean age: 36 years, range: 21–65) participated. The volunteers were asked about infections, skin diseases, and their personal assessment of their skin sensitiveness. Subjects who had used antibiotics or other immune-modulating medications less than 8 weeks prior to the study, or had current abnormal discharges, like itching or irritations, were excluded. Also hospitalised persons or health care workers were excluded. The subjects gave their written consent to inclusion. All participants were under dermatological supervision. The sampling areas were checked for any irritating shift weekly over a period of 6 weeks, starting at the outset and ending 1 week after the wear period.

### 2.2. Textile Samples

The wear trial was planned as a placebo-controlled right/left-intraindividual pre-/post-comparison with special T-shirts. These were constructed in 2 separate halves, both with the same look. One side consisted of a nonantibacterial 100% polyester knitted fabric (placebo, Interlock 42E, dtex 76 f 128), whereas the other half was knitted with an antibacterial 100% polyester yarn endowed with silver (verum 1, Trevira bioactive, Bobingen, Germany, the mass area per unit was 146 g/m^2^). The placebo and verum fabrics were combined using press buttons in the front and back and worn during the night at least for 8 h over a period of 4 weeks. After each week, new T-shirts were used and the worn samples were washed and checked for their antibacterial activities (>3 log step was required). Halves were washed separately (according to DIN EN ISO 6330: 2001–2004 Textiles—Domestic washing and drying procedures for textile testing, at 40°C with 9.5°dH and 78.5 g ECE detergent) in order to avoid antibacterial contamination of the placebo fabrics. 

Another antibacterial fabric (verum 2) was made by finishing nonantibacterial single jerseys made of Polyamide-Tactel (dtex 85 f 92/Linel 33, mass area per unit was 147 g/m^2^) by a common padding method using a commercially available antibacterial finish (Beisoft-SH, CHT Beitlich, Tübingen, Germany). In addition, an alcohol-free, silver-containing antibacterial deodorant was used for a one-day short-term wear trial (verum 3: deodorant). 

To exclude any secondary skin irritation effects, all fabrics and deodorants were checked prior to the wear trial for their cytotoxic and irritating potential according to EN ISO 10993 Biological evaluation of medical devices, Part 5: tests for *in vitro* cytotoxicity and Part 10: tests for irritation and delayed-type hypersensitivity.

### 2.3. Antimicrobial Activity

The antibacterial activities of all textile samples were evaluated with the suspension test according to the standard ISO 20743:2007 “Textiles—determination of antibacterial activity of antibacterial finished products.” The determination of the antibacterial activity was performed by the absorption method, in which a test bacterial suspension is inoculated directly onto samples. In brief, textile swatches were inoculated with a starting suspension of 10^5^ of *Staphylococcus aureus* and *Klebsiella pneumoniae*. After 18 h incubation at 37°C, the colony plate count method was used for the enumeration of bacteria colony forming units (CFUs). The specific antibacterial activity was determined by inoculating a negative control material of the same sort of fabric but without the antibacterial activity. The efficiency of the activity was then calculated by the following equation:

(1) $$\begin{array}{cc} & {\text{Log}}_{10}\text{CFU}\;\left(\text{negative}\;\text{control},\;18\;\text{h}\right)\\ & \;\;\;-{\text{Log}}_{10}\text{CFU}\;\left(\text{sample},\;\;18\;\text{h}\right)\\ & \;\;=\text{specific}\;\text{antibacterial}\;\text{activity}. \end{array}$$

The general assessment criteria follow a definition by Hohenstein Institutes, in that a growth reduction efficacy of <0.5 corresponds to no antibacterial activity, whereas ≥0.5 to <1 corresponds to a slight, ≥1 to <3 to a significant, and a growth reduction of ≥3 indicates a strong antibacterial activity, respectively.

### 2.4. Silver Release

The silver release of the textile swatches (1 gr) was determined by shaking the textiles for 24 h in artificial sweat solution according to DIN EN ISO 105-E04: 1996–2008 Textiles—Tests for colour fastness—Part E04: Colour fastness to perspiration (0.5 g L-Histidine-Hydrochloride monohydrate, 5 g NaCl, 2.2 g Na-dihydrogenphosphate, pH 5.5) at 37°C. Two spray bursts of the deodorant were also collected in sweat solution. The silver release of the sweat solutions was determined by ICP-MS (Elan 9000, Perkin-Elmer, Germany).

### 2.5. Skin Microflora Examinations

Bacterial solutions were collected weekly from the back of the participants, at the region of their scapula of both body sides, immediately before the wear trial (baseline, T_0_), during the trial (T_7_, T_14_, T_21_, T_28_) as well as 7 days thereafter (T_35_). The scapular region was chosen because it ensures that all textiles had a close fit and was permanently covered with the sample. The participants were evaluated always by the same investigator. A standard scrub method developed by Williamson-Kligman [18] was used to collect the skin microflora samples from the two back sides. Sterile glass cylinders (2.0 cm in inner diameter with a contact area of 3.14 cm^2^) were placed on the scapula skin. 2 mL of sterile Phosphate-Buffered Saline (containing 137 mM NaCl, 2.7 mM KCl, and 10 mM phosphate, pH 7.4) were poured into the cylinder followed by continuous scrubbing of the surface with a blunt sterile rubber policeman for 20 seconds. The liquid was removed with a sterile pipette and emptied into a sterile 15 mL test tube. 100 *μ*L each were plated on blood agar plates. 

Plates were incubated at 36°C under aerobic conditions and inspected after 2 days. The number of colony forming units (CFUs)/cm^2^ was determined. Using routine bacteriological techniques microorganisms were categorized into coagulase-negative staphylococci (CNS), *Staphylococcus aureus*, *Streptococci* spp., *Micrococcus* spec., Bacilli, enterobacteria, Gram-positive rods (propionibacteria, corynebacteria), yeasts and the total number of aerobic microorganisms. The analytical data were expressed as logarithms for CFU per cm² of skin.

### 2.6. Statistics

The Wilcoxon ranked pair test was applied for comparison of the colonization on antibacterial active verum sides versus the nonantibacterial placebo sides in comparison to baseline at different time points of evaluation. A significance level of *P* = .05 was chosen. Means and standard deviations were calculated by means of SigmaStat 3.5 and the Wilcoxon-Signed-Rank Test.

### 2.7. Skin Physiology Parameters

To analyse possible influences of antibacterial clothes to the skin, an objective quantification of skin parameters was performed by measuring hydration (skin capacitance), evaporimetry/transepidermal water loss (TEWL), and pHmetry, bilaterally, on the scapular region. Test persons stayed in a conditioned room with 22°C and 40% RH during 15 min to acclimatize the skin. The TEWL, as an indicator of the stratum corneum integrity, was measured with an evaporimeter (g/m²∗h; Tewameter TM 300, Courage and Khazaka Electronics, Köln, Germany). Electrical capacitance, as an indicator of the stratum corneum hydration (corneometry) was measured in triplicate with a capacitance meter. For this purpose, a Multiprobe Adaptor System MPA-9 (Courage and Khazaga, Cologne, Germany) was used, equipped with Corneometer CM 825 and a pH 905 pH-meter. According to the manufacturer, the pHmeter measures with a precision of 0.1 unit and a punctual (pointed) electrode. The probe was preheated to skin temperature, and the measured TEWL values were recorded over a period of 60 seconds. In addition the skin temperature was measured with an infrared thermometer (Dostmann Electronic, Wertheim-Reichbolzheim, Germany).

## 3. Results

Prior to the wear trials, the antibacterial activities of the textile samples and the deodorant were determined. The polyester verum fabric 1 reduced the starting inoculum of 10^5^ germs over 4.25 log steps for *S. aureus *and also showed a very strong activity against *K. pneumoniae* (3.38 log-step reduction). The finished antimicrobial T-shirt half displayed a somewhat lower activity of 3.03 log-step reductions for *S. aureus *and a strong 4.24 log-step reduction for *K. pneumoniae*. Thus, the antibacterial activities of the fabrics were in efficacy levels typical for sport and leisure wear. Silver releases, determined by ICP/MS, showed a release of 2.5 ppm of silver from the verum 1, whereas the finished verum 2 released 1.9 ppm of silver. In contrast to this, the silver content of the deodorant amounted 0.25 ppm, although it exhibited strong antibacterial activities versus both test germs. The results are summarized in Table 1. 

**Figure 1:**
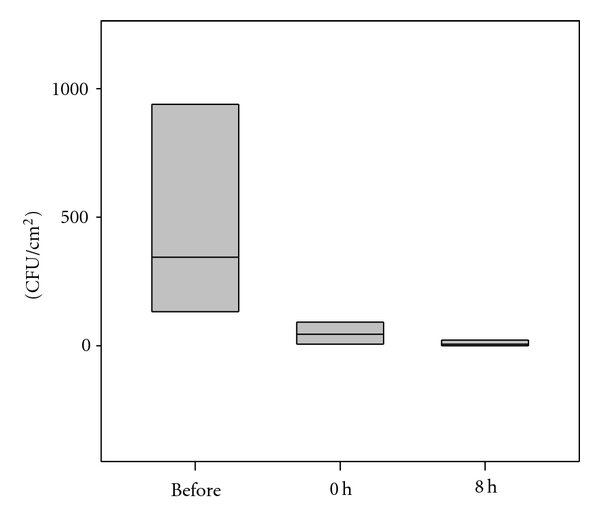
Boxplot diagram showing a short-term impact on the microflora immediately (0 hour) and after 8 hours following a spray burst of an antibacterial silver-containing deodorant (*n* = 8).

To evaluate a possible short-term impact on the physiological human skin microflora, a spray burst of an antibacterial silver-containing deodorant was applied on the scapula of the volunteers. Immediately after the application and 8 h later, skin bacteria were recovered, counted, and compared to the number of skin germs before the spray application. The before and after line boxplot is given in Figure 1. A significant drop of microorganisms was observed immediately after the application (0 h), which lasted at least more than 8 h, indicating a short-term impact on the microflora. The microflora recovered to normal values after 24 h (not shown). The spectrum of germs did not change.

**Figure 2:**
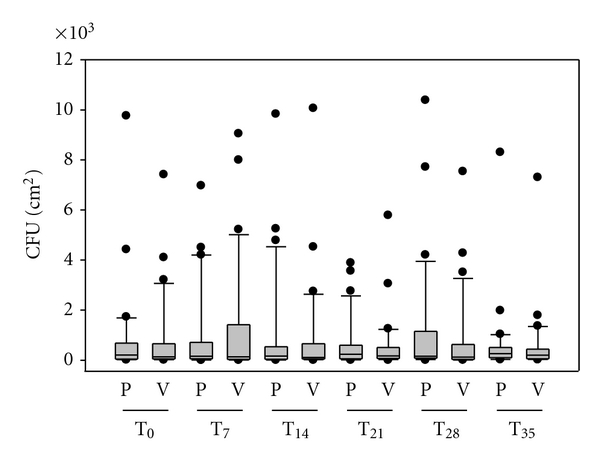
Boxplot diagram showing total germ count after application of fabric 1. PES-silver Verum side (V) and placebo side (P). T_0_ = baseline, T_7_ = after 1 week wear trial, T_14_ = after 2 weeks, T_21_ = after 3 weeks, T_28_ = after 4 weeks, T_35_ = 1 week after the wearing time (*n* = 30).

**Figure 3:**
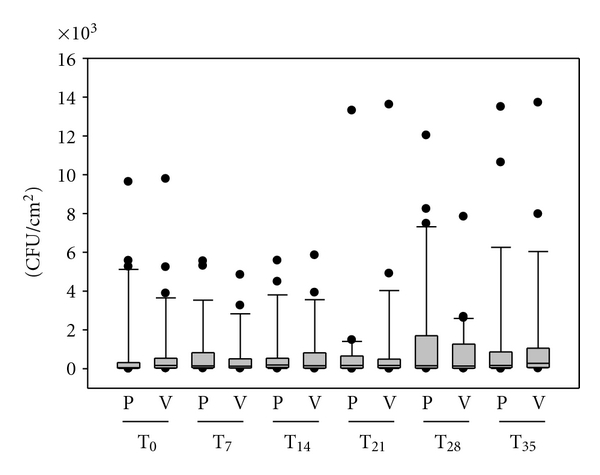
Boxplot diagram showing total germ count after application of fabric 2. Silver-finish Verum side (V), placebo side (P). T_0_ = baseline, T_7_ = after 1 week wear trial, T_14_ = after 2 weeks, T_21_ =after 3 weeks, T_28_ = after 4 weeks, T_35_ = 1 week after the wear period (*n* = 30).

The placebo-controlled side-to-side comparison with special T-shirts was run, to evaluate possible long-term effects on the ecological balance of the human skin flora of antimicrobial clothes. For both fabrics, the halves knitted with silver-loaded fibres as well as for the halves finished with silver, the effects on the human skin microflora were identical. Figures 2 and 3 summarize the results. At all measuring points we analyzed typical bacteria of the human microflora. No pathogenic germs occurred in the microflora of the subjects during the wear period or afterwards. Furthermore, no significant deviations were found for the total cell counts of the body side covered by the antibacterial half of the T-shirt, or the side covered with the control material of similar structure. In the box-whisker blot, the interquartile ranges (IQRs) of all volunteers were similar. On each body side, the spectrum of microorganisms did not change during the wear trial. 

**Figure 4:**
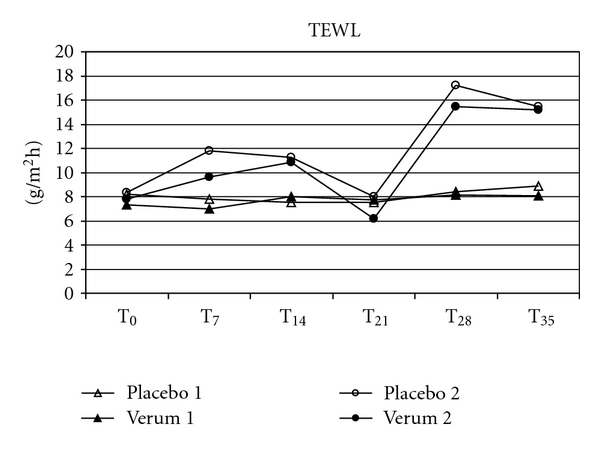
The comparison of the mean values of TEWL between the antibacterial verum and the corresponding placebo shirt halves did not show significant differences between the baseline (T_0_), during (T_7_–T_28_) or after the wear trial (T_35_).

**Figure 5:**
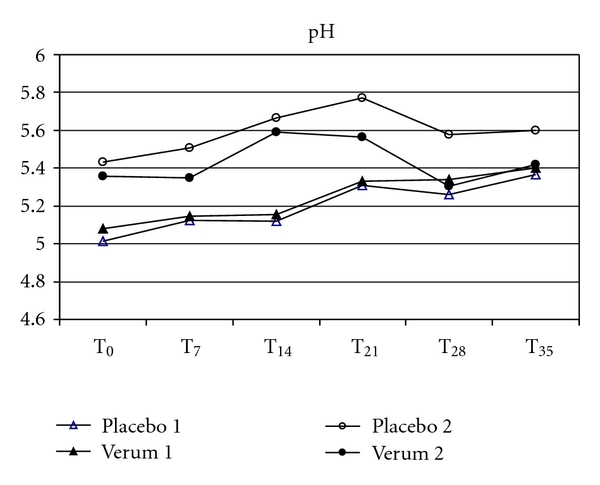
The comparison of the mean values of skin pHmetry between the antibacterial verum and the corresponding placebo shirt halves did not show significant differences between the baseline (T_0_), during (T_7_–T_28_) or after the wear trial (T_35_).

**Figure 6:**
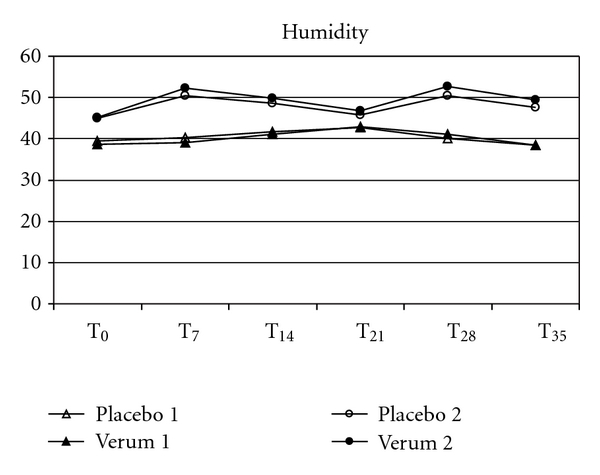
The comparison of the mean values of skin hydration between the antibacterial verum and the corresponding placebo shirt halves did not show significant differences between the baseline (T_0_), during (T_7_–T_28_) or after the wear trial (T_35_).

Possible secondary effects on the skin were determined by measuring the skin physiological parameters pH-value, TEWL and hydration in order to look for a changing microbial composition of the skin microflora or the advent of irritations. Skin parameters were taken prior to each skin scrubbing, that is, before, weekly and a week after the removal of the T-shirts (Figures 4, 5, and 6). Minor individual right/left deviations were observed, which remained in parallel over the wear period. None of the test subjects' skin pH values showed a deviation of  more than 0.5 at each weekly measuring point or after the wear trial, when compared to the individual pH value prior to the wear trial. There was also no significant difference for TEWL or the skin moisture over the measuring period as compared to the corresponding placebo sides. Furthermore, over the whole test period of six weeks, the sampling areas were checked weekly by a dermatologist for any irritating shift. None of the test persons showed signs for skin irritations or allergies within the test area.

## 4. Discussion

Although wearing clothing to protect one's skin is not a new practice, there are limited investigations on physiological responses of skin towards clothes. Parameters like water and water-vapour transport through garments have already been shown to influence microclimate and subsequently the flora of the skin [19], but, to our knowledge, there are only scarce data available on microflora properties, when the skin gets into contact with antibacterial fabrics [20]. Studies are therefore needed to understand the chemical and biological effects of antimicrobial textiles on skin health.

### 4.1. Antibacterial Activity, Release, and Risks of Silver

Today, metallic silver and silver compounds are predominantly used in antibacterial fabrics by contrast with other biocides, that is, in sport and leisure wear as well as in fabrics for the treatment of atopic dermatitis [3, 6–8, 11]. As the use of silver products increases, it is becoming more important to develop standard procedures to measure the efficacy of each product in order to discuss questions concerning comparability, mechanisms and risks [21]. For example, various dermatological studies propagating the efficacy of silver cloth in lowering *S. aureus* colonization of atopic dermatitis patients failed to use standard procedures to measure the antibacterial efficacy of their samples, neglecting the fact that an industrial key standard (ISO 20743:2007) exists to determine the antibacterial activity of antimycrobially finished products [22]. Technically, the setup of this suspension tests enforces a close proximity and the interaction of test germs with the surface of the antibacterial fibres. Therefore, the resulting log-reduction values, for example, cannot be compared to standardized testing of disinfectants and antiseptics. As a result, the standard ISO 20743 favours high log reductions of antibacterial fabrics, notably when there is only a slight release of biocides. Against this background, we found *strong* activities for the silver-loaded and the silver-finished shirt halves according to the general assessment criteria.

For an alternative assessment of the antibacterial activity, we also measured the silver release of our samples. The total silver release of the silver-loaded fabric was 2.5 ppm, whereas the silver-finished shirt released 1.9 ppm. This is slightly above the minimum of 1 ppm, which is considered to be required for an antimicrobial activity [11] and supports the view that the ISO 20743 favours high log reductions. Nevertheless, compared to silver-loaded wound dressings, which have been shown to release around 10–40 ppm of silver, the silver release of commercially sport and leisure wear is far lower [12, 23]. For many silver-containing products, in contrast to antibiotics, the minimal inhibition concentration (MIC) values and breakpoints of silver still have not been agreed by professional organizations [13, 24, 25]. To complicate matters further, other factors beside the release, for example, the distribution of silver within a product, its chemical and physical forms, also influence its ability to kill microorganisms [21]. Concerning the risk of silver fabrics, the release of approx. 2 ppm of silver exhibits low toxicity in the human body and minimal risk is expected due to dermal application, inhalation, ingestion or through the urological or haematogenous route [10–12]. A further risk factor of silver is its allergenic potential. Silver allergy is discussed as a contraindication for using silver in antibacterial clothes, although the incidence of this rare allergy is still not known [10]. Therefore, despite seldom silver allergies, silver-containing medical devices are evaluated as safe in the use on patients.

### 4.2. Effects on the Microbiome

The main objective of this pilot study was to investigate whether antibacterial clothes affect the skin microbiome. To prove possible effects on the ecological balance of the healthy human skin flora, we performed long-term wear trials with form-fitting clothes and also compared the effects to the short-term application of an antibacterial deodorant. 

Recent studies employing 16SrRNA gene survey strategies with samples from the inner elbow of healthy human subjects indicate that the human skin microbiome is far more complex and in fact comprises 113 phylotypes that belong to six bacterial divisions [26]. Gene survey analyses are unsuitable for field trials, although it can be assumed that the skin microbiome on the healthy scapular skin and of atopic skin is of similar diversity [27]. *In vivo* studies for testing the efficacy of topical antimicrobial agents require the evaluation of the skin flora by more easy-to-perform methods. Since fabrics may only contact the skin's surface yet for a limited period (due to bending stiffness and drapeability), we used a standard scrub method for the recovery of typical aerobic skin bacteria from the scapula, which allows a satisfying enumeration and identification [18, 28]. The analysis by culture-dependent assays also allowed to distinguish between viable and nonviable bacteria. 

Over a wear trial of six weeks, the skin flora was analyzed weekly for opportunistic and pathogenic microorganisms on healthy human skin. No pathogenic germs occurred in the microflora of the subjects during the wear period of four weeks or afterwards. Furthermore, no significant deviations were found for the total cell counts of the body side covered with the antibacterial half of the T-shirts or the side covered with the control material of similar structure. On each body side, the spectrum of microorganisms did not change during the wear trial. Thus, neither the T-shirts with antibacterial silver-finish nor silver-loaded fibres disturbed the skin flora in number or composition. In contrast to this experiment, one single disinfection of the scapula region with an alcohol-free silver-based deodorant of similar strong activity caused a short-term reduction of 2 log-steps in the total germ count.

Our results support the view that the human skin microflora is quite stable towards exogenous destabilisation which is interesting for a risk assessment of antibacterial clothes used for staffs, working in health care institutions (e.g., in nursing homes, intense care units, paramedics), where the antibacterial fibre surface may have beneficial means of controlling life-threatening nosocomial infections including MRSA as well as adding levels of personal hygiene [29]. Lilly et al. concluded that the eradication of the bacterial population of the normal skin flora is impossible *in vivo*, even after repeated applications of skin disinfectants [30]. On the other hand, the constant and excessive use of antimicrobials is known to cause irritant and allergic contact dermatitis [14]. Furthermore, changes in the bacterial flora may occur: they are often associated with skin damages, infections, frequent showering or use of skin care products, hence, hands of health care personnel are often affected [31]. We did not find opportunistic microorganisms on our healthy volunteers, indicating that antibacterial clothes do not impair the colonization resistance of healthy skin. This is in line with Cole et al. [32], who investigated the antibiotic and antibacterial agent cross-resistance in target bacteria from homes of antibacterial product users and nonusers. They showed that the use of antibacterial products does not facilitate the development of antibiotic resistance in bacteria from the home environment.

### 4.3. Fabrics in Dermatotherapy

Many lines of evidence suggest a role for microorganisms in noninfectious skin diseases, such as atopic dermatitis, rosacea and acne [33, 34] Therefore, antiseptic therapies using liquid skin disinfectants and systemic antimicrobials are essential for the efficient dermatotherapy of affected lesions of atopic dermatitis patients [3]. Hartmann [35] has shown that liquid antimicrobials may have a short-term impact on the ecosystem of the skin flora, a view which is supported by our results taken with the antibacterial silver deodorant. These impacts are of clinical importance, when members of the normal skin flora are involved in the pathogenesis of the disease, for example, *Propionibacterium acnes* in acne vulgaris, *Corynebacterium* species in erythrasma, *S. aureus* in atopic dermatitis and others. 

The clinical efficacy of adding an antimicrobial effect to fabrics in the treatment of atopic dermatitis patients has been investigated by many research groups [6–8]. Gauger et al. [3] also used a side-to-side comparative trial by comparing the treatment with silver-coated textiles on one arm to that of cotton on the other arm for 7 days followed by 7 days without the treatment in 15 patients with generalized or localized atopic dermatitis. In contrast to our results taken over 4 weeks, their study demonstrated a highly significant decrease in the nonphysiological *S. aureus *colonization on the side covered by the silver-coated textile already after 2 days. It was furthermore concluded, that overnight wearing might be able to sustain a constant *S. aureus* reduction. Mason already noted that the mechanisms on the eradication of *S. aureus* on atopic skin are unclear [36], in particular against the background of the lack of testing the antibacterial efficacy (see above). In contrast to our study the textiles used by Gauger were form fitting to the skin, and all patients were allowed to use topical steroids. Moreover, atopic dermatitis patients have sensitive and impaired skin barrier functions [37]. We were unable to find *S. aureus* colonization on the skin of our healthy subjects. Nevertheless, despite these differences, antibacterial textiles may play an important clinical role, especially in skin conditions with an increased rate of bacterial or fungal infections like atopic dermatitis and hyperhidrosis, in diabetic patients or aged skin [38].

### 4.4. Microclimate

The interactions between fabrics and skin climate and their impact on the skin microflora have already been studied by Runeman et al. [19]. The temperature, pH, and total number of microorganisms were significantly lower for users of vapour-permeable panty liners. In this study we monitored skin barrier functions depending on antibacterial cloth by measuring TEWL, stratum corneum hydration and skin surface pH, in order to look for the advent of irritations or secondary effects of a changing microbial composition of the skin microflora. We were unable to find significant difference for TEWL, pH, or skin moisture over the whole measuring period for the antibacterial shirt halves as compared to the corresponding placebo sides. Since resident microbiota may become pathogenic, sometimes in response to an impaired skin barrier [17], our results speak in favour of no effect on skin barrier and occurrence of pathogenic bacteria. This is also supported by our finding that the sampling areas were checked weekly by a dermatologist for any irritating shift. None of the test persons showed signs for skin irritations or allergies within the test area.

## 5. Conclusions

Altogether, in our experiments we were not able to see any significant adverse effects of antibacterial clothes on the physiological human skin microflora or the skin barrier of healthy people. Worth of note is that the subject of evaluation was healthy skin that is already in good conditions at the start of the study.

## Figures and Tables

**Table 1:** Antibacterial activities and silver release of the tested textiles and the deodorant.

	Activity (log cfu) *Staphylococcus aureus *	Activity (log cfu) *Klebsiella pneumoniae *	Ag-release (ppm)
Verum 1	4.25	3.38	2.5
Verum 2	3.03	4.24	1.9
Deo	2.95	4.72	0.25
